# Retraction: L-Plastin Nanobodies Perturb Matrix Degradation, Podosome Formation, Stability and Lifetime in THP-1 Macrophages

**DOI:** 10.1371/journal.pone.0320874

**Published:** 2025-03-13

**Authors:** The PLOS One Editors

After this article [1] was published, concerns were raised regarding results presented in Figs 2, 4, 5, and S2.

Specifically:

- The following results appear similar when color levels are adjusted:
  ◦ Fig 2B,
    - LPL Nb5 Nb-EGFP CL panel, LPL Nb5 Nb-EGFP -Dox panel, and LPL Nb9 Nb-EGFP CL panel.
    - EGFP EGFP CL panel and EGFP EGFP -Dox panel.
    - GSN Nb11 Nb-EGFP CL panel and GSN Nb13 Nb-EGFP CL panel.
  ◦ Fig 2C LPL Nb5 Actin blot and Figure 2C CapG Nb4 Actin blot.
- The following results appear similar despite representing different experimental conditions:
  ◦ Fig 4D Pro-MMP9 MMP9 EGFP CapG Nb4 panel and Figure 4D Pro-MMP9 MMP9 GSN Nb11 GSN Nb13 panel when flipped horizontally.
  ◦ Fig S2A lanes 3 and 6.
  ◦ Fig S2B lanes 1–2 and S2B Figure lanes 5–6 when flipped horizontally.
- There appear to be vertical or horizontal discontinuities in the following panels:
  ◦ Fig 2B G.S.N. Nb11 GSN +Dox
  ◦ Fig 2B G.S.N. Nb13 GSN +Dox
  ◦ Fig 4C between lanes 1 and 2, and between lanes 4 and 5.
  ◦ Fig 4E between lanes 2 and 3.
  ◦ Fig 5B G.S.N. Nb11 Phospho-LPL, L.P.L. Nb5 Phospho-LPL, L.P.L. Nb5 LPL, LPL Nb9 Phospho-LPL and L.P.L. Nb9 LPL
  ◦ Fig S2A between lanes 4 and 5.

The authors did not respond to editorial requests for a response and underlying data. In light of the above unresolved concerns that question the integrity and reliability of the reported results and conclusions, the *PLOS One* Editors retract this article.

All authors either did not respond directly or could not be reached.
